# The Users’ Intention to Participate in a VR/AR Sports Experience by Applying the Extended Technology Acceptance Model (ETAM)

**DOI:** 10.3390/healthcare10061117

**Published:** 2022-06-15

**Authors:** Kyongmin Lee, Seungwook Oh

**Affiliations:** 1 Department of Sport Industry, Korea National Sport University, Seoul 05541, Korea; dsukmlee77@outlook.kr; 2 Department of Sport BIGtainment, Kyung Hee University, Seoul 17104, Korea

**Keywords:** VR/AR sports experience, extended technology acceptance model, experience economy factors, presence, flow

## Abstract

Since the basic model of technology acceptance model (TAM) was introduced, studies in various fields have been conducted to improve the explanatory power of the TAM. These studies show that there are limitations in explaining the intention to use a new technology with just two variables (perceived usefulness and perceived ease of use), and that the intention to use a new technology can be affected by many external variables, in addition to the two variables. Accordingly, the purpose of this study is to identify factors affecting users’ intention to participate in a virtual/augmented reality (VR/AR) sports experience based on the extended technology acceptance model (ETAM). To this end, this study analyzed the results of a survey conducted on 300 university students who are sports majors in Korea, who have experienced VR/AR sports. First, among experience economy factors, education experience, escape experience, and esthetic experience had a positive effect on presence. Second, presence had a positive effect on perceived usefulness and flow. Third, perceived usefulness and flow had a positive effect on the intention to participate. This study has significance in empirically investigating the relationship among variables affecting users’ intention to participate in a VR/AR sports experience using the ETAM. The results can be used as basic data for establishing strategies necessary in vitalizing VR/AR sports in the future.

## 1. Introduction

Recently, indoor sports using virtual/augmented reality (VR/AR) technology are receiving attention due to the constraints on outdoor sports activities caused by the COVID-19 pandemic or health, weather, and environmental factors such as heat waves or particulate matter [1]. The VR/AR sports experience refers to sports activities using VR and AR technology. With the development of immersive VR/AR sports technology, a virtual sports experience that had been limited to screen golf is expanding into other sports, such as baseball, soccer, and t-ball, enabling a similar experience to actual sports activities by interacting with the virtual world in reality [2].

Global market research company P&S Market Research [3] estimated that the global VR/AR market size will increase by approximately 3263%, from $37.9 billion in 2019 to $1.2744 trillion in 2030, as VR/AR technology is adopted more widely in various fields such as games, entertainment, education, and rehabilitation following neuromuscular injury. The VR/AR sports market, is also expected to constantly grow beyond simple experience-based content, with the growing interest in the digital world and sports convergence on the internet and the increasing need to develop VR/AR platforms for training and physical education to improve athletic performance [2].

As the market for VR/AR sports is constantly growing, studies have been conducted on improving policies and systems to vitalize VR/AR sports [4,5,6], remedying and correcting technical issues [7], using VR/AR sports in physical education [8,9], and promoting VR sports facility management [10,11]. These studies have significance in providing policy, technological, and marketing implications necessary for vitalizing VR/AR sports, but they are limited in explaining factors from the user perspective that affect the acceptance and intention to participate in VR/AR sports. Accordingly, this study identifies factors affecting users’ intention to participate in a VR/AR sports experience using the extended technology acceptance model (ETAM).

Since the basic technology acceptance model (TAM) was introduced, studies have been conducted in various fields to increase the explanatory power of the TAM. These studies are classified into those investigating antecedent factors (e.g., technological characteristics, subjective characteristics of users, social characteristics, etc.) affecting perceived usefulness and perceived ease of use [12,13,14], and those applying additional variables (e.g., trust, pleasure, hedonic motivation) aside from these two key variables [15,16,17]. These studies show that there are limitations in predicting the intention to use a new technology with just these two variables (perceived usefulness and perceived ease of use), and that the intention to use a new technology can be affected by many external variables aside from perceived usefulness and ease of use.

Therefore, the purpose of this study is to empirically investigate the relationship among the variables affecting user acceptance and the use of VR/AR sports experiences using the ETAM. More specifically, it uses perceived usefulness and flow as determinants of the intention to participate in a VR/AR sports experience and analyzes the relationship among the variables. Next, as external variables of perceived usefulness and flow, this study selects presence that is a VR/AR media factor, and experience economy that is a media user factor, to analyze the relationship among the variables. Finally, it analyzes the relationship between experience economy and presence.

This study has theoretical implications in that it extends the predictive power of the TAM by applying the TAM to VR/AR sports and presents an extended TAM showing the process of users’ participation in VR/AR sports experience by adding external variables that may affect acceptance and use of VR/AR sports experience.

## 2. Theoretical Background and Hypothesis Setting

### 2.1. VR/AR Technology and Technological Limitations

VR and AR technology is referred to as immersive media in that VR/AR technology provides users with an immersive experience [18]. VR technology creates a virtual world that is not real but similar to reality using computer technology, and AR technology embodies a world where virtual objects and information made of computer technology are combined with the real world [18]. Both VR and AR create virtual images using computer technology, but AR overlays a virtual image on a real environment, while VR provides the virtual image in a virtual environment instead of reality [18]. As shown in Table 1, this technological difference makes the distinction between the core technologies that represent VR and AR [19].

Since VR/AR technology can be widely applied to daily life such as entertainment, education, medical care, and sports, the economic and social ripple effect is expected to be enormous in the future. However, technological problems such as visual discomfort and heavy weight still pose obstacles to the popularization of the VR/AR market [20]. Visual discomfort is caused by dynamic errors. Dynamic error refers to a time delay that occurs in the process of executing a virtual image corresponding to a change in the user’s gaze [20]. When a dynamic error occurs, an error occurs between the real environment and the virtual environment that the user sees, causing the user to feel visual discomfort. In addition, HMD devices, used when using VR/AR content, are so heavy that users feel uncomfortable wearing them [20]. Therefore, to activate VR/AR technology, technical supplement is required to reduce dynamic errors and lighten the weight of HMD.

### 2.2. Experience Economy Factors of VR/AR Sports Experience and Presence 

Experience economy is classified into four factors such as entertainment experience, esthetic experience, education experience, and escape experience, depending on special events, individual participation level, and connection [21]. Entertainment experience is when individuals experience hedonic feelings such as pleasure or fun through special events. This refers to the case in which individuals observe the event passively instead of making or creating it [22]. In this study, entertainment experience is defined as pleasure or fun experienced by users during VR/AR sports experience. Esthetic experience is when the esthetic elements of facilities or settings of a special event space affect personal emotions [21]. This is similar to entertainment experience in that consumer participation is passive, but it is different in that individuals can experience it when immersed in the environment [22]. In this study, esthetic experience is defined as interior decorations, indoor lighting, and experiential content design inside the VR/AR sports venue, experienced by users during the experience.

Education experience is when individuals obtain new knowledge and information through special events and experience things related to new knowledge [21]. During an education experience, individuals experience new things by actively participating in the special event while keeping a certain distance [23]. Education experience in VR/AR sports indicates obtaining knowledge or skills related to sports through sports experience. Escape experience is when individuals escape or deviate from their daily routines and enjoy themselves through special events [21]. Individuals experiencing escape tend to participate actively in the event in perfect flow [23]. Escape experience in VR/AR sports indicates that users feel the joy of escaping from their daily routines by being immersed in a sports experience.

Presence is defined as the extent to which users feel as if they actually exist in the virtual space created by the medium [24], the extent to which users feel as if they are moving into the virtual world provided by the medium [25], and the subjective mental state in which users feel that the space, objects, and characters in the virtual world created by the medium actually exist in reality [26]. In summary, presence refers to the subjective feeling of users thinking that, even though the virtual world experience implemented by a certain medium is created by the medium’s technology, the technological elements of the medium that enable the experience of presence itself do not really exist [18]. Therefore, presence in this study is defined as the extent to which users are convinced that they actually exist in the virtual environment during a VR/AR sports experience.

There are various factors affecting the presence of users in a virtual environment created by the medium, the determinants of presence can be largely classified into technological factors and user factors of the medium [27]. For example, users can experience higher presence in the medium providing functions such as convenience in use [27], big screens [28], and high definition and high sound quality [25]. Moreover, prior experience in using a specific medium [29], user gender [30], and motivation or engagement [31] can be classified as antecedent factors affecting the presence of users in a medium environment. In this context, the technological features provided by VR and AR, and users participating in a VR/AR sports experience can be the key variables for the formation of presence.

Users have extraordinary experience when they enjoy or have fun in a VR/AR sports experience or reach the state of flow by actively participating and having a meaningful experience that adds value to their life [32]. This is expected to help enhance the subjective experience through which users are convinced that they actually exist in a virtual space during VR/AR sports experience.

Studies on the relationship between experience economy and presence in a VR/AR environment are as follows: Jung, Dieck, Lee, and Chung [33] discovered a positive relationship between VR and AR experience of museum visitors (entertainment, escape, esthetic, and education experience) and presence. Kim and Park [34] also claimed that tourism experience (entertainment, escape, esthetic) through AR applications has a positive effect on presence. Accordingly, this study set up the following hypotheses based on the results of previous studies:

**Hypothesis** **1** **(H1).** *Factors of VR/AR sports experience economy (entertainment, education, escape, esthetic) will have a positive effect on the presence of users.*

**Hypothesis** **1a** **(H1a).** *Entertainment experience will have a positive effect on presence.*

**Hypothesis** **1b** **(H1b).** *Education experience will have a positive effect on presence.*

**Hypothesis** **1c** **(H1c).** *Escape experience will have a positive effect on presence.*

**Hypothesis** **1d** **(H1d).** *Esthetic experience will have a positive effect on presence.*

### 2.3. Presence, Perceived Usefulness, and Flow

Perceived usefulness is known to be one of the key antecedent variables that affects user attitude in the process of accepting new technology [35]. In other words, users who perceived high usefulness of new technology due to certain external factors may have a higher intention to continue using the technology [35]. In this study, perceived usefulness is defined as the extent to which users believe that VR/AR sports experience is useful.

In general, the more users are convinced that they exist in a virtual space created by the medium, the more they believe that the medium is useful. Kwon and Wen [36] discovered that users who experienced a high level of telepresence in a social media environment highly perceive the usefulness of SNS (Social Network Service) use, and Oum and Han [37] proved that telepresence had a significant effect on perceived usefulness in a study about acceptance of user-created contents service. A study by Wei and Li [38], on acceptance of mobile learning, also revealed that telepresence is an antecedent factor of perceived usefulness. Accordingly, this study set up the following hypothesis based on the results of previous studies:

**Hypothesis** **2** **(H2).** *Experience of presence in VR/AR sports experience will have a positive effect on perceived usefulness.*

Flow is the optimal mental state experienced when an individual is fully concentrating on a certain activity. Individuals experience the state of flow when they have a clear goal to achieve from a certain activity, can provide quick feedback on the activity, and perceive that the activity gives them a high level of challenge and that they have high skills to handle the activity [32]. In the state of flow, individuals fully concentrate on the ongoing activity and believe that they have control over it, thereby taking charge of the activity. They also completely forget the time flow and have inner satisfaction through which they ultimately feel pleasure and enjoy the activity itself [32]. In this study, flow is defined as the state in which users are fully concentrating on VR/AR sports experience.

In a VR and AR environment, a high presence of users may lead to a high level of flow [39,40,41], which is why presence was selected as a factor affecting the flow of users. In an AR-based learning environment, enhancing presence promotes learning flow by providing a real learning experience [39], and self-presence of virtual sports experience users played a key role in increasing flow [40]. Kim and Jeon [41] also proved that higher telepresence of tourist site AR leads to higher user flow. Accordingly, this study set up the following hypothesis based on the results of previous studies.

**Hypothesis** **3** **(H3).** *Experience of presence in VR/AR sports experience will have a positive effect on flow.*

### 2.4. Percievied Usefulness, Flow, and Intention to Particpate

Intention to participate has been used as a key variable to explain whether individuals continue using certain technology in studies applying the ETAM, since it directly affects actual individual behavior [42,43,44]. In other words, a higher intention to participate promotes users to continue using certain technology, which may also lead them to actively recommend the technology to others [45]. In this study, intention to participate is defined as the intention to actively and continuously use a VR/AR sports experience.

In previous studies applying the ETAM, perceived usefulness had a positive effect on the intention to participate. Moon and Byun [42] studied Offline to Online (O2O) food service participants and discovered that higher perceived usefulness of the O2O food service led to a higher intention to participate, and Park, Lee, and Kim [43] proved that the perceived usefulness of home workout equipment had a positive effect on users’ intention to continue to use it. Min [44] also revealed the causal relationship between the usefulness of contactless fitness training and continuous use by participants. Accordingly, this study set up the following hypothesis based on the results of previous studies.

**Hypothesis** **4** **(H4).** *Perceived usefulness will have a positive effect on users* *’ intention to participate in VR/AR sports experience.*

The concept of flow is recently used in predicting users’ intention to use new media in a digital media environment. Studies on the mobile game environment [46], sports video game environment [47], IT (Information Technology)-based home workout environment [48], and online broadcasting [49] confirmed that there is a significant positive relationship between users’ flow experience and their intention to continue using the media. The results of these studies show that the intention to continue participating in a VR/AR sports experience may be affected by users’ flow experience. Accordingly, this study set up the following hypothesis based on the results of previous studies.

**Hypothesis** **5** **(H5).** *Flow will have a positive effect on users* *’ intention to participate in VR/AR sports experience.*

## 3. Research Method

### 3.1. Research Subjects

To test the hypotheses, this study selected samples using convenience sampling among sports majors currently enrolled in a four-year university degree course located in a capital area of Korea, who have experience doing sports activities at a VR/AR sports experience center. A total of 290 out of total 300 copies of the questionnaire were collected, and 262 copies, excluding 28 copies with insincere responses, were ultimately used in the statistical analysis. Table 2 shows the demographic characteristics of the samples.

### 3.2. Measurement Tool

This study used the questionnaire including items to measure experience economy factors, presence, perceived usefulness, flow, and intention to participate, along with the demographic characteristics of the subjects. All items excluding the demographic characteristics were rated on a 5-point Likert scale. Table 3 shows formation and source of the measurement tool.

Items on experience economy factors were measured using the tool used by Kim and Park [34], revised to be more suitable for this study, based on items used in the studies by Park and Yoon [50], Oh, Fiore, and Jeoung [51], and Pine and Gilmore [52]. There were 12 items related to experience economy factors: 3 items on entertainment experience, 3 items on education experience, 3 items on escape experience, and 3 items on esthetic experience.

A total of 4 items were related to presence, selected based on the questionnaire developed by Kim and Park [34] that was revised to be more suitable for this study, with reference to the study by Inma and Antoni [53].

Perceived usefulness was measured using the questionnaire used by Noh and Choi [54] and Lee and Park [55], revised to be more suitable for this study. Perceived usefulness was a single factor measured using a total of 3 items.

Items on flow were measured with the tool used by Kim and Jeon [41], revised and supplemented to be more suitable for this study, based on items used by Csikszentmihalyi [56] and Privette and Bundrick [57]. Flow was a single factor measured using a total of 3 items.

Finally, items on the intention to participate were measured using the tool used by Oh, Lee, An, and Moon [58], revised and supplemented to be more suitable for this study, based on items used by Barclay, Higgins, and Thompson [59], Donath and Boyd [60], and Steinfield, Ellison, and Lampe [61]. Intention to participate was a single factor measured using a total of 3 items.

Once the questionnaire was developed, a panel of experts was formed to evaluate whether the questionnaire included the concept to be measured, and whether each item was appropriate. The experts included full-time faculty members and doctoral students in sport management programs.

### 3.3. Analysis Method

Collected data was statistically analyzed using SPSS 21.0 (IBM, Armonk, NY, USA) and AMOS 21.0 (IBM, Armonk, NY, USA), and confirmatory factor analysis was conducted to test the validity of the measurement tool. Moreover, Cronbach’s α, representing the internal consistency of items, was calculated to test the reliability of the tool. Correlation analysis was conducted to determine the correlation among variables, and structural equation modeling was performed to test the hypotheses.

### 3.4. Validity and Reliability of the Measurement Tool

Table 4 shows the results of testing the fit of the model. As a result of conducting the confirmatory factor analysis on each factor, the model fit indices were *χ²* = 377.062 (*df* = 247, *p* < 0.001), *χ*²/df = 1.527, TLI = 0.949, CFI = 0.958, and RMSEA = 0.045, thereby meeting the standard (Song, 2015). All values of construct reliability (CR) and average variance extracted (AVE) exceeded 0.70 and 0.50, and, as shown in Table 5, r² among all variables was smaller than AVE. Therefore, the scales used to measure the variables in this study had adequate convergent and discriminant validity [62]. Moreover, Cronbach’s *α* coefficients were also 0.625–0.872, all exceeding 0.6, that is, the threshold of reliability assessment, proving that the internal consistency among items is generally showing a satisfying level of reliability [62].

## 4. Results

### 4.1. Correlation Analysis

Table 5 shows the correlation among the variables included in this study. The correlation among factors was positive, and the correlation coefficient of all factors was lower than *r* = 0.8, indicating that there is no multicollinearity problem among variables. Moreover, AVE among variables was higher than the square root of the correlation coefficient, thereby securing discriminant validity [62].

**Table 5:** Correlation analysis among variables.

Variable	1	2	3	4	5	6	7	8
Entertainment experience	1 (0.708)							
Education experience	0.596 **	1 (0.722)						
Escape experience	0.611 **	0.555 **	1 (0.673)					
Esthetic experience	0.256 **	0.283 **	0.220 **	1 (0.696)				
Presence	0.499 **	0.576 **	0.489 **	0.364 **	1 (0.728)			
Perceived usefulness	0.160 **	0.155 *	0.181 **	0.133 *	0.331 **	1 (0.790)		
Flow	0.175 **	0.248 **	0.220 **	0.244 **	0.352 **	0.264 **	1 (0.767)	
Intention to participate	0.174 **	0.220 **	0.131 *	0.170 **	0.242 **	0.321 **	0.406 **	1(0.738)

** *p* < 0.01. * *p* < 0.05, ( ) = AVE.

### 4.2. Hypothesis Testing

Table 6 shows the results of testing the research model using a structural equation. The fit of the model was *χ*^2^ = 389.868 (*df* = 261, *p*<.001), *χ*^2^/df = 1.494, TLI = 0.952, CFI = 0.958, and RMSEA = 0.043, which is relatively favorable and thus has no problem with hypothesis testing.

Entertainment experience did not have a significant effect on presence (*β* = 0.108, *p* = 0.309), and thus, hypothesis 1a was rejected. Education experience had a significant positive effect on presence (*β* = 0.343, *p* < 0.001), and thus, hypoethsis 1b was accepted. Escape experience had a significant positive effect on presence (*β* = 0.255, *p* < 0.05), and thus, hypothesis 1c was accepted. Esthetic experience had a significant positive effect on presence (*β* = 0.255, *p* < 0.01), and thus, hypothesis 1d was accepted. Therefore, hypothesis 1 was partially accepted. Presence had a significant positive effect on perceived usefulness (*β* = 0.376, *p* < 0.001) and flow (*β* = 0.423, *p* < 0.001), and thus, hypotheses 2 and 3 were accepted. Perceived usefulness had a significant positive effect on intention to participate (*β* = 0.283, *p* < 0.001), and thus, hypothesis 4 was accepted. Flow had a significant positive effect on intention to participate (*β* = 0.415, *p* < 0.001), and thus, hypothesis 5 was accepted.

## 5. Discussion

The purpose of this study was to empirically analyze how the presence of VR/AR media affects users’ intention to participate in a VR/AR sports experience based on the ETAM. To this end, this study examined the causal relationship between experience economy factors and presence, and verified the relationship of presence with the perceived usefulness and flow that are selected as determinants of the intention to participate in a VR/AR sports experience. Moreover, it examined the effect that perceived usefulness and flow have on users’ intention to participate in a VR/AR sports experience. Based on the results, the following discussions can be made.

### 5.1. Summary of Findings

First, education experience, escape experience, and esthetic experience, among factors of VR/AR sports experience economy, had a positive effect on presence, whereas entertainment experience did not have an effect. These results are partially consistent with the findings of previous studies [33,34], which have asserted that experience economy factors play a major role in increasing presence. Second, experience of presence in a VR/AR sports experience had a positive effect on perceived usefulness and flow. This is consistent with the results of previous studies claiming that presence is an important determinant of perceived usefulness [36,37,38], and presence affects flow [39,40,41]. Third, perceived usefulness and flow had a positive effect on the intention to participate. This is in line with the studies claiming that perceived usefulness affects the intention to participate [42,43,44] and that flow is a key antecedent variable for the intention to participate [46,47,48,49].

### 5.2. Theoretical Implications

#### 5.2.1. Relationship between VR/AR Sports Experience Economy and Presence

First, presence increased when users had education, escape, and esthetic experience while participating in VR/AR sports, while entertainment experience did not affect presence. Witmer and Singer [63] mentioned flow as a determinant of users’ experience of presence in a virtual environment created by the media. Flow is the state in which individuals are deeply concentrating on specific activities, and individuals in the state of flow fully concentrate on the activity they are carrying out, and are thereby completely absorbed in the specific activity while being oblivious to external stimulations [32]. Escape experience occurs when the levels of participation and flow of participants and special events are high, and esthetic experience occurs when the level of participation is low, but flow is high [21]. Therefore, when users have an escape experience and esthetic experience while participating in VR/AR sports, they are completely immersed in the VR/AR sports content and are, thus, more convinced that they are actually playing the sports in the virtual environment.

Users also experienced presence in an education experience while participating in VR/AR sports. Education experience occurs when the level of flow is low and the level of participation is high [21]. In a study related to AR games, Han [64] classified the level of participation into interest and ease of use, and proved the positive causal relationship between level of participation and presence. The results of this study showed that certain media users can experience a high level of presence when they are interested in activities embodied in the virtual environment and perceive that it does not require much effort to use the media.

In this respect, when participants had an education experience while playing VR/AR sports, they could not be completely immersed in the sports content created by VR/AR, but they may have experienced the presence of the virtual sports space created by the media by paying attention and actively participating in the content. Moreover, the subjects of this study are sports majors who have more interest in experiencing VR/AR sports than students with other majors. They also might not have felt much difficulty or inconvenience in obtaining knowledge or skills related to the sports through VR/AR technology, which may have increased the presence of university students.

Unlike education, escape, and esthetic experience, the entertainment experience did not play a significant role in forming presence of VR/AR sports experience users. In this study, entertainment experience is defined as the fun and pleasure experience by users when participating in VR/AR sports experience. Entertainment experience related to participation in VR/AR sports does not require active participation and high flow of users [21], and thus did not lead to a high level of presence.

#### 5.2.2. Relationship of Presence with Perceived Usefulness and Flow

Users perceiving higher presence tend to show higher perceived usefulness and flow. In this study, the causal relationship of presence with perceived usefulness and flow can be explained by the media characteristics of VR/AR. VR/AR quickly and promptly provides users with abundant information and clues to experience the virtual environment through various sensory organs, in addition to visually rendering the virtual environment. Thus, users are likely to experience a higher level of presence in a virtual environment based on VR/AR, compared to other media [18,39]. Due to the media characteristics of VR/AR that provide all kinds of information and sufficient clues that excite the senses, users feel as if the sports they play in the virtual space are real [18,39], which led to higher flow and perceived usefulness of users in a sports experience in the virtual environment.

#### 5.2.3. Relationship of Perceived Usefulness and Flow with Intention to Participate

Users with higher perceived usefulness and flow in a VR/AR sports experience showed a greater intention to continuously and actively use VR/AR sports. According to the motivation theory, factors that motivate individual behavior can be classified into intrinsic and extrinsic motivation based on self-determination [65]. Intrinsic motivation is carrying out a particular activity just for the joy of it, not for extrinsic rewards, whereas extrinsic motivation is doing it to obtain certain outcomes (e.g., monetary reward, social status) rather than the joy of the activity itself [66]. Therefore, users’ flow in a VR/AR sports experience is a form of intrinsic motivation, while perceived usefulness is a form of extrinsic motivation. Users immersed in VR/AR sports experience are intrinsically motivated, completely absorbed and enjoying the activity itself [32]. People with intrinsic motivation are drawn to the joy of the activity itself and are thus likely to continue participating in the activity [67]. In this respect, users’ intention to participate in VR/AR sports activities is likely to increase when they experienced flow in a VR/AR sports experience.

People with extrinsic motivation are motivated by extrinsic rewards they may earn when they attain their goal rather than the activity itself, which is why they carry out the activity only when they perceive that it would be helpful [68]. Therefore, users who perceived that VR/AR sports activities are useful, participate in VR/AR sports activities because of extrinsic rewards such as improved athletic ability and skills, rather than the joy of participating in the activity itself.

### 5.3. Practical Implications

The results of this study can help establish strategies necessary to vitalize the VR/AR sports experience by identifying antecedent variables that may affect users’ intention to participate in a VR/AR sports experience.

First, in this study, users’ perceived usefulness and flow in a VR/AR sports experience had a positive effect on their intention to participate. This shows that it is necessary to enhance users’ perceived usefulness and flow in order to vitalize a VR/AR sports experience. For users to reach the state of flow in which they are fully immersed in the contents during VR/AR sports experience, they must be able to experience positive emotions that are fun and enjoyable [32]. Therefore, developers of VR/AR sports experience content must develop and supply sports content services that offer all kinds of interesting attractions. To attract users whose motivation to participate in VR/AR sports activities is usefulness, it is necessary to publicize that the VR/AR sports experience may actually help relieve stress, and that a sports experience similar to reality can be gained at a relatively reasonable price.

Next, this study shows that it is necessary to promote the presence of users in a VR/AR sports experience so that they can fully concentrate on the VR/AR sports experience and perceive that the experience is useful. Technical attributes in the media-based virtual environment can be a key determinant for the formation of presence among users [25,27]. However, since presence is subjective experience of media users convinced that they actually exist in the media-based virtual environment [18], practical discussions on media user factors that may affect presence can also be important, aside from the technical attributes to promote presence of media users.

Education, escape, and esthetic experience were among the factors of the VR/AR sports experience economy selected, as media user factors had a positive effect on the increase in presence, whereas entertainment experience did not have any effect. This shows that there is a need for VR/AR sports experience content in which users can be immersed and actively participate, in addition to just being fun and interesting in order to increase the presence of users in a virtual environment. It is necessary to create content through which users can actively obtain and improve knowledge and skills related to sports and to design attractive content. Furthermore, there is also a need to pay attention to the interior decorations and indoor lighting within the experience center so that users can concentrate on the VR/AR sports experience.

## 6. Conclusions

The significance of this study can be summarized in three aspects. First, this study provided an extended model that can explain users’ acceptance and intention to participate in a VR/AR sports experience by adding presence and flow, which are VR/AR media characteristics, to the conventional TAM. Second, this study selected experience economy factors, which are media user factors, as antecedent variables for increasing users’ presence in a VR/AR sports experience, and empirically investigated the causal relationship between the two variables. Finally, the results of this study provided strategies to vitalize the VR/AR sports experience.

The limitations of this study and suggestions for future studies are as follows: First, university students majoring in sports were selected as the subjects of this study, assuming that university students have much VR/AR sports experience. However, it is difficult to consider that university students accurately represent the entire population using a VR/AR sports experience. Therefore, future studies must empirically examine whether similar results are obtained in other age groups. Additionally, the user profile is very important in an immersive environment, since any particularity can change the type of perceived experience. Thus, qualitative analysis can be used to determine the detail of the VR/AR sports experience itself with other age groups.

Second, according to the TAM, perceived ease of use, aside from perceived usefulness, also serves as a key determinant that affects user attitudes affecting the intention to accept new technology [35]. However, in this study, perceived ease of use was not included as an antecedent factor affecting users’ intention to participate in a VR/AR sports experience. This is due to the assumption that the subjects of this study would not have much difficulty or inconvenience in using a VR/AR sports service. However, in many studies applying the TAM [12,13,69], perceived ease of use was proven to be a key determinant for the intention to accept and continue using new technology. Therefore, future studies must include perceived ease of use in the model presented in this study and validate the effect of the variable. Moreover, many other variables that may affect acceptance and continuous use of a VR/AR sports experience may also be added to increase the explanatory power of the TAM.

## Figures and Tables

**Table 1:** VR/AR core technologies.

Technology		Explanation
VR	Immersion visualization	Technology that provides uses with an immersive virtual reality environment Visualization device technology such as HMD (head mounted display), projection, image visualization technology software
	Relistic interaction	Technology that corresponds to input/output between VR participants and systems based on the user’s five senses Motion-based simulator, VR participant location tracking, tactile, haptic, olfactory, and taste-reated technologies
	Virtual reality environment creation and simulation	Technology that creates a VR enviornment based on 360 degree panoramic images or restoration Technology that provides scenario-based immersion visualization and interactive environment for VR partipants
AR	Sensing and tracking	Tehcnology that postions virual objects in real space for augmentation precisely Technology that supports tracking of new spaces
	Image synthesis	Technology that expresses virual objects in line with images in real space Device technology that provides an AR enivronment to users and technology that semalessly aynthesizes images with real space
	Real-time AR interaction	Technology that allows users to experience AR space through real-time interaction with virtual objects synthesized in real space (e.g., Pokemon Go)

Note. Reprinted from “VR·AR·MR related technology and policy trends” by H. Yoon [19], p. 11.

**Table 2:** General characteristics of subjects.

Category		Frequency	Percentage
Gender	Male	161	61.5
	Female	101	38.5
Year	Freshman	50	19.1
	Sophomore	90	34.4
	Junior	67	25.6
	Senior	55	21.0
Age	Under 21	52	19.8
	21 to 22	91	34.7
	23 to 24	75	28.6
	25 and above	44	16.8

**Table 3:** Formation and source of the measurement tool.

Factor	Variable	Number of Item	Source
Experience economy	Entertainment experience	3	Kim and Park [34], Park and Yoon [50], Oh, Fiore, and Jeoung [51], Pine and Gilmore [52]
	Education experience	3	
	Escape experience	3	
	Esthetic experience	3	
Presence		4	Kim and Park [34], Inma and Antoni [53]
Perceived usefulness		3	Noh and Choi [54], Lee and Park [55]
Flow		3	Kim and Jeon [41], Csikszentmihalyi [56], Privette and Bundrick [57]
Intention to participate		3	Oh, Lee, An, and Moon [58], Barclay, Higgins, and Thompson [59], Donath and Boyd [60], Steinfield, Ellison, and Lampe [61]
Total		25	

**Table 4:** Results of confirmatory factor analysis and reliability analysis.

Item	Esti.	S.E.	C.R.	CR	AVE	α
Entertainment experiences						
The VR/AR sports experience was fun and enjoyable	1			0.878	0.708	0.824
The VR/AR sports experience provided various interesting attractions	1.284	0.111	11.609			
The VR/AR sports experience made me feel joy	1.208	0.108	11.232			
Education experiences						
The VR/AR sports experience satisfied my curiosity	1			0.885	0.722	0.850
The VR/AR sports experience improved knowledge or skills related to the sports	1.338	0.099	13.491			
The VR/AR sports experience helped learn new sports skills	1.235	0.098	12.638			
Escape experience						
I felt as if I were in a different world during the VR/AR sports experience	1			0.859	0.673	0.817
Time flew by during the VR/AR sports experience	0.892	0.073	12.258			
I could forget about my daily routines during the VR/AR sports experience	0.881	0.075	11.784			
Esthetic experience						
The content design of VR/AR sports experience was attractive	1			0.867	0.696	0.625
The indoor lighting of the VR/AR sports experience space was adequate	1.024	0.146	7.011			
The interior decorations of the VR/AR sports experience space were attractive	0.514	0.097	5.281			
Presence						
After VR/AR sports experience, I felt as if I came back to reality	1			0.914	0.728	0.793
During the VR/AR sports experience, the world inside the media was realistic	1.169	0.124	9.399			
During the VR/AR sports experience, I felt as if I was right inside the world	1.273	0.129	9.857			
A new world opened up to me as I started the VR/AR sports experience, which suddenly disappeared the moment I stopped	1.025	0.118	8.656			
Perceived usefulness						
The VR/AR sports experience allowed me to experience the same feeling at a relatively lower price than a real-life experience	1			0.918	0.790	0.872
The VR/AR sports experience actually helps my life, such as relieving stress	1.007	0.073	13.868			
The VR/AR sports experience helps improve athletic ability and skills	1.155	0.082	14.095			
Flow						
Using the VR/AR sports experience fulfilled my expectations	1			0.907	0.767	0.830
I lost track of time during the VR/AR sports experience	1.252	0.107	11.713			
I was completely immersed in the content during the VR/AR sports experience	1.034	0.092	11.181			
Intention to participate						
I will put in a lot of time in a VR/AR sports experience	1			0.894	0.738	0.837
I will continue participating in a VR/AR sports experience	1.182	0.098	12.122			
I will actively participate in a VR/AR sports experience	1.138	0.096	11.896			

**Table 6:** Structural model analysis results.

Hypothesis Testing	Estimate		S.E.	C.R.
	B	*β*		
H1a entertainment experience → presence	0.095	0.108	0.093	1.017
H1b education experience → presence	0.281	0.343	0.070	4.033 ***
H1c escape experience → presence	0.171	0.255	0.069	2.488 *
H1d esthetic experience → presence	0.199	0.223	0.062	3.226 **
H2 presence → perceived usefulness	0.650	0.376	0.128	5.100 ***
H3 presence → flow	0.509	0.423	0.096	5.298 ***
H4 perceived usefulness → intention to participate	0.193	0.283	0.045	4.268 ***
H5 flow → intention to participate	0.406	0.415	0.072	5.670 ***

*χ*^2^ = 389.868(*df* = 261, *p* < 0.001), *χ*^2^/df = 1.494, TLI = 0.952, CFI = 0.958, RMSEA = 0.043; * *p* < 0.05. ** *p* < 0.01. *** *p* < 0.001.

## Data Availability

Not applicable.
